# Amino Acid Nutrition and Metabolism in Health and Disease

**DOI:** 10.3390/nu11112623

**Published:** 2019-11-01

**Authors:** Adam J. Rose

**Affiliations:** Nutrient Metabolism & Signalling Laboratory, Department of Biochemistry and Molecular Biology, Metabolism, Diabetes and Obesity Program, Biomedicine Discovery Institute, Monash University, Clayton 3800, Australia; adam.rose@monash.edu

**Keywords:** amino acid, nutrition, metabolism, dietary protein restriction, supplements, exercise, D-amino acids

## Abstract

Here an overview of the special issue “Amino acid nutrition and metabolism in health and disease” is given. In addition to several comprehensive and timely reviews, this issue had some original research contributions on fundamental research in animal models as well as human clinical trials exploring how the critical nutrients amino acids affect various traits.

Of the three major macronutrients, protein is critical for vitality [1]. Protein can be digested and absorbed as amino acids [2] and short peptides [3], both of which have important somatic effects. Amino acids are the building blocks of our cellular machinery in the form of proteins and protein complexes. In addition, many important metabolites (i.e., purine/pyrimidines, neurotransmitters etc.) are products of cellular amino acid metabolism. In contrast to that of fats (lipid droplets in adipose tissue) and carbohydrates (glycogen in liver and muscle), there is no dedicated storage depot of protein in the body, and body protein is conserved during periods of total nutrient deficiency or withdrawal [4]. This is achieved by cellular mechanisms such as recycling of amino acids via autophagy and processing of proteins within the lysosome [5], as well as physiological processes such as the dampening cellular turnover of amino acid consuming tissues [5], the inhibition of protein synthesis in quiescent tissues [6,7], and fine control of ureagenesis matched to dietary protein supply [8]. Nevertheless, some important biochemical pathways require that the amino acid amine group is lost and thus there is then an obligatory loss of amino acids which needs to be replaced via food consumption [9]. Thus, adequate protein nutrition is paramount to supply such indispensable amino acids [10]. In this special issue on “Amino Acid Nutrition and Metabolism in Health and Disease” we highlight new information as well as review several important topics within this sub-field of nutrition.

Preclinical studies have shown that dietary protein restriction can promote metabolic health [11,12,13,14,15,16]. In their paper, Javed and Bröer [17] show that mice lacking the neutral amino acid transporter B^0^AT1 have a serum amino acid profile resembling that of mice fed a low protein diet. Given that B^0^AT1 is the major transporter of neutral amino acids across the intestinal lumen and reabsorbs neutral amino acids in the renal proximal tubules [18], this reinforces that the inhibition of this transporter might be an attractive strategy to mimic the effects of dietary protein restriction to improve health and retard age-related disease [19]. 

Branched-chain amino acids (BCAA) have been implicated as a major contributor to the effects of dietary protein supply on metabolic health [20,21]. In their paper, Ribeiro et al. [22] assessed and directly compared the relationship between circulating BCAAs, body composition, and intake in older mice and men. They found that protein intake was correlated with circulating BCAA levels, and that body weight and body fat were positively associated with circulating BCAA levels, in both mouse and human. In their paper, David et al. [23] examined the relationship between circulating BCAA levels, body composition and tissue BCAA catabolic capacity in rats made insulin resistant by high-fructose feeding. In this model, the high BCAA levels in insulin-resistant rats were not associated with differences in body composition, but were correlated with altered skeletal muscle BCAA catabolic capacity.

The dietary restriction of sulphur containing amino acids (SCAA), in particular L-methionine and L-cysteine, confer health benefits to age-related disease [24]. The regulation and role of signaling pathways, particularly the integrated stress response, was reviewed in detail by Jonsson et al. [25]. They highlight a disconnect between canonical integrated stress response signaling and adaptive responses to SCAA restriction. In addition, two original contributions from clinical trials addressing the role of SCAA were published in this special issue. One by Olsen et al. [26] reported results of a pilot randomized clinical trial testing the combination of dietary SCAA restriction and high unsaturated fatty acid supply on feasibility and certain AA biomarkers, and another by Lee et al. [27] examined the relationship between acute and chronic exercise, insulin sensitivity, and plasma amino acid levels. The latter study [27] provided evidence that both acute and long-term exercise may influence trans-sulphuration and glutathione biosynthesis, and suggested a link between exercise-improved insulin sensitivity and oxidative stress/mitochondrial function.

There were further contributions of clinical trials investigating select amino acid supplements on traits. Of note, Tsuda et al. [28] studied the effects of combined L-arginine, L-valine and L-serine supplementation on exercise-induced fatigue in healthy volunteers using a randomized, double-blinded, placebo-controlled crossover design. They demonstrated that supplementation with the amino acid mixture reduced the feeling of fatigue during exercise. It will be interesting to see if such results affect actual exercise performance in future studies. On L-arginine, Hsu & Tian [29] reviewed the role of L-arginine synthesis and metabolism in pregnancy, with a focus on developmental programming of non-communicable diseases. They presented an overview of emerging evidence from experimental studies showing that targeting the L-arginine metabolic pathway has promise as a reprogramming strategy in pregnancy to prevent non-communicable diseases in the offspring. With the premise that the amino acids L-arginine and L-citrulline can affect nitric oxide, a well-described vasodilatory substance [30], Khalaf et al. [31] reviewed the effects of these amino acids on blood pressure regulation. They provided evidence that oral L-arginine supplementation can lower blood pressure to a comparable extent to that of exercise and diet interventions, effects that are not well understood and perhaps deserve greater attention, particularly as deaths and burden due to hypertension rival that of cancer [32,33].

On cancer, Bastings et al. [34] provided an intriguing review of the various sources of D-amino acids, their metabolism, and their contribution to physiological processes and diseases, with a focus on cancer. Once considered to be non-functional or not even present in living organisms, these enantiomeric counterparts of L-amino acids are now acknowledged to play important roles in numerous physiological processes in the human body.

In addition to the paper by Ribeiro et al. [22], other contributions also examined the role of dietary protein/amino acid supply in age-related disease. As menopause is associated with a spike in age-related disease incidence in women [35], Lin et al. [36] examined the potential beneficial effects of soy protein supplementation and exercise training on ‘postmenopausal’ mice. They found that a combination of soy protein supplementation and exercise reduced fatigue and improved bone function in ovariectomized mice. 

With our ageing population, age-related dementias are a prominent health issue. In their review, Glenn et al. [37] described the current understanding of dietary protein and amino acids and the preventative roles they play with regard to age-related dementias, and provide future directions to follow for this field.

Even though there are several cellular pathways such as GCN2 and mTORC1 which signal amino acid availability with appropriate cellular responses and fates, peptide hormones also play an equally important role in conveying dietary amino acid availability with traits and behaviors. On this topic, Rose [38] reviewed the regulation and roles of certain peptide hormones in response to altered dietary protein supply, with focus on glucagon, PYY and FGF21.

Going forward, the future is bright concerning the topic of protein/amino acid nutrition in health and disease, and particular promising directions—such as inter-organ amino acid nutrition, tissue/niche heterogeneity of amino acid metabolism, and the role of the microbiome and epigenome in the interaction of amino acid supply/metabolism with various traits such as immunity—should be considered for future study. Indeed, with the advent and application of new technologies and ‘big data’, many new interesting and pivotal interactions will undoubtedly be uncovered.
